# Supplementary material for: Impact of Gut Microbiota and SCFAs in the Pathogenesis of PCOS and the Effect of Metformin Therapy
Source: Int J Mol Sci. 2024 Oct 2;25(19):10636. doi: 10.3390/ijms251910636 (PMC11477200; doi:10.3390/ijms251910636)
Supplement: Supplementary file 1 [file ijms-25-10636-s001.zip › Supplementary_S1.pdf]

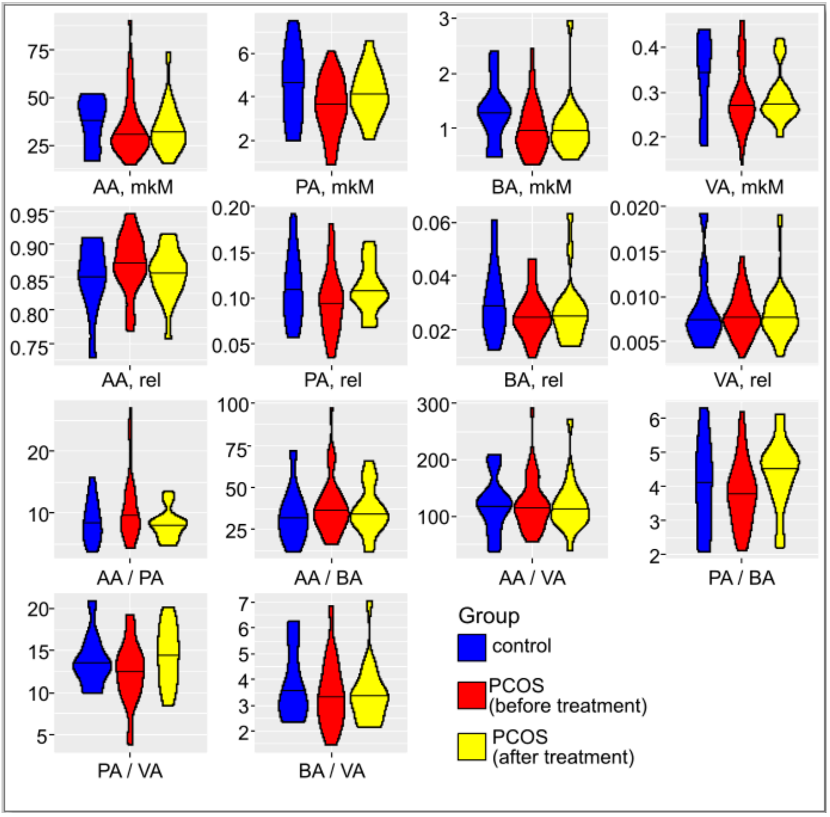

Figure S1. Concentrations of SCFAs in serum. Blue - control group (n=10), red – PCOS group before treatment (n=38) and yellow – PCOS group after treatment (n=69). AA - acetic acid, VA - valeric acid, BA - butyric acid, PA - propionic acid.

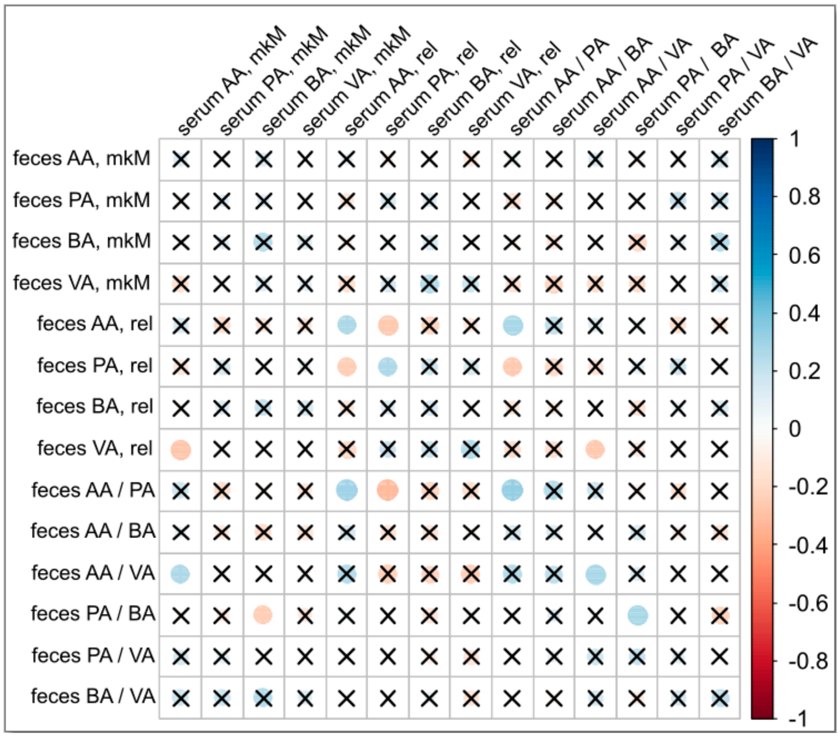

Figure S2. Correlation of serum versus fecal SCFA levels.

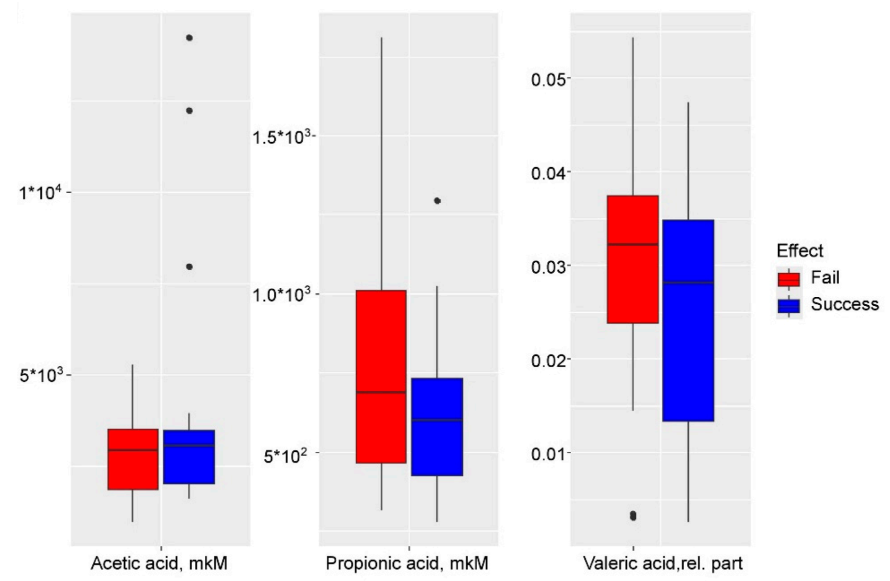

Figure S3. Fecal SCFA signatures which were included in XGboost model for predicting the full therapeutic effect. * - statistically significant alteration according to Mann-Whitney test. Blue color – full effect, red color – no effect of the metformin's therapy.

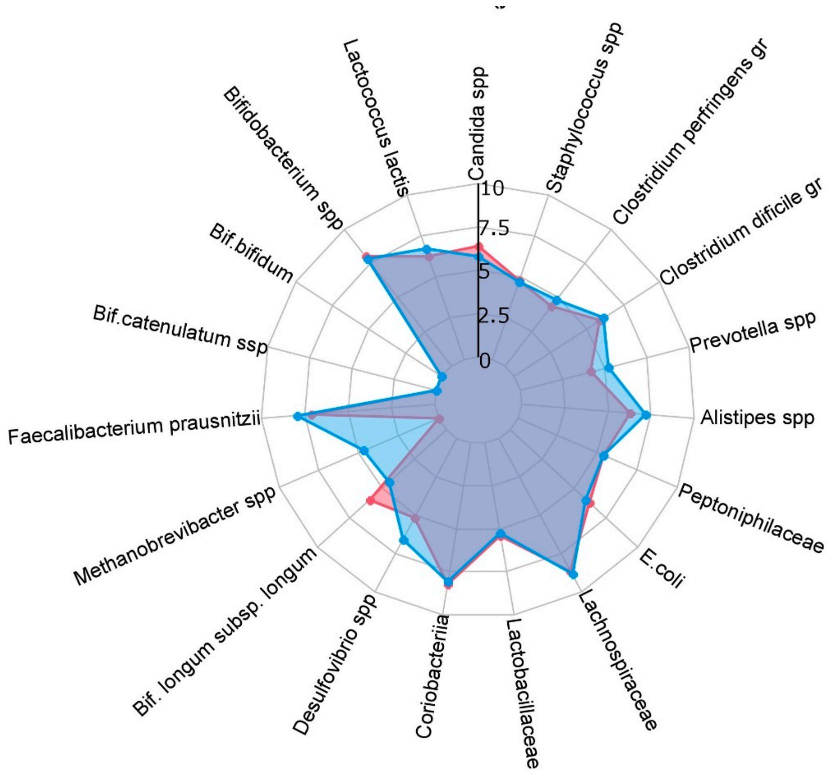

Figure S4. Radio chart of microbiota features (logarithmic scale) which were included in XGboost model for predicting the full therapeutic effect. * - statistically significant alteration according to Mann-Whitney test. Blue color – full effect, red color – no effect of the metformin's therapy.

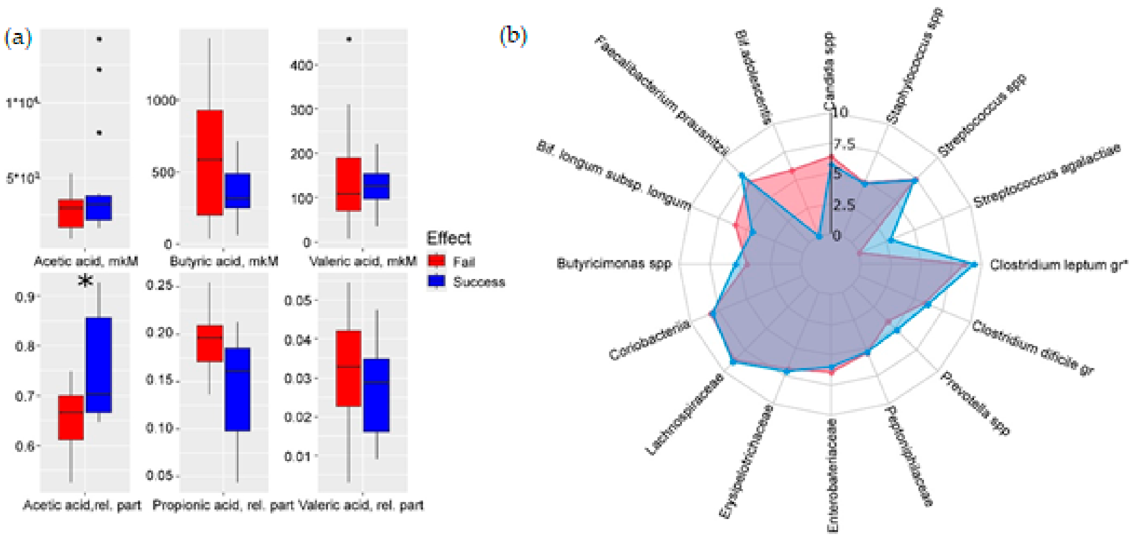

Figure S5. XGboost model for predicting the full therapeutic effect based on the fecal microbiota and SCFAs. (a) Fecal SCFA signatures which were included in model. (b) Radio chart of microbiota features (logarithmic scale) which were included in the model. * - statistically significant alteration according to Mann-Whitney test. Blue color – full effect, red color – no effect of the metformin's therapy.

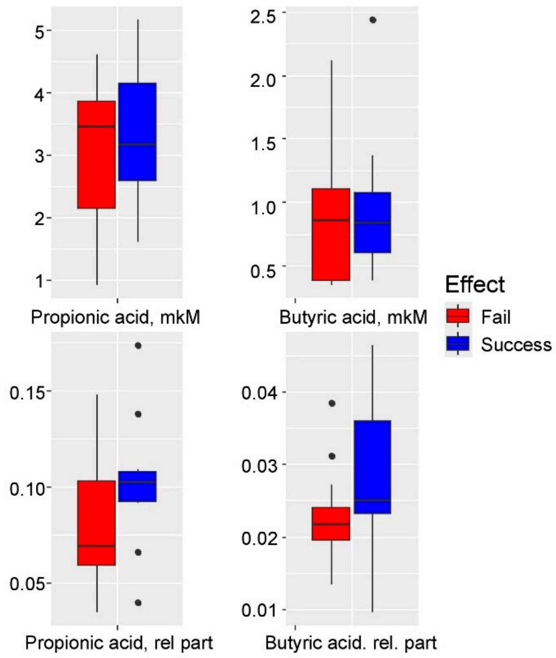

Figure S6. XGboost model for predicting the full therapeutic effect based on the serum SCFAs. * - statistically significant alteration according to Mann-Whitney test. Blue color – full effect, red color – no effect of the metformin's therapy.

Figure S7. Calibration curves for SCFAs in feces. Milli-Q water was used as matrix. Black circle – standard, green square – IS, blue rectangle – QC.

1 – Acetic acid

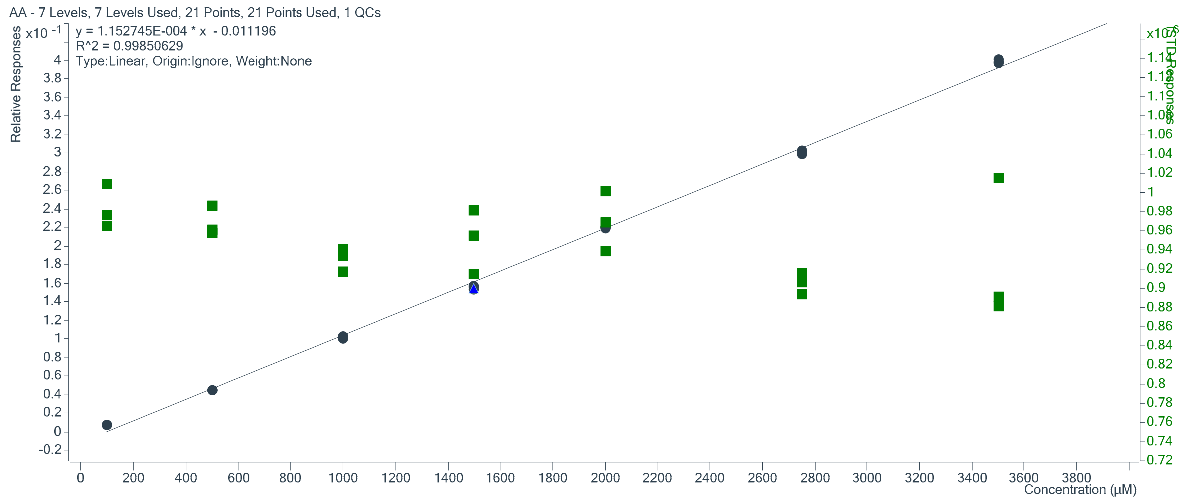

2 – Propionic acid

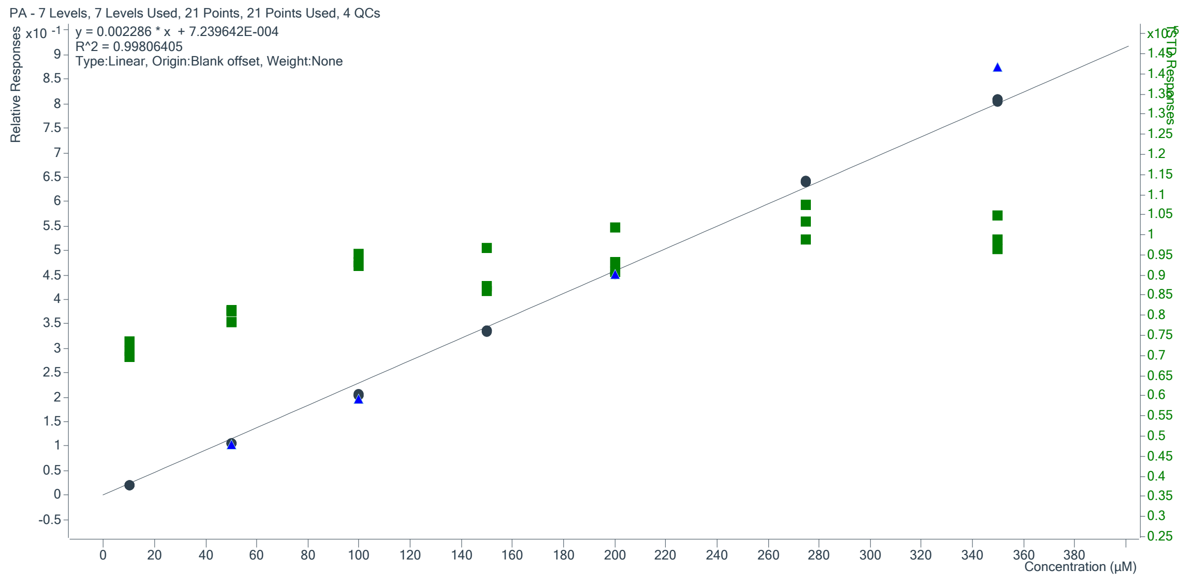

3 – Butyric acid

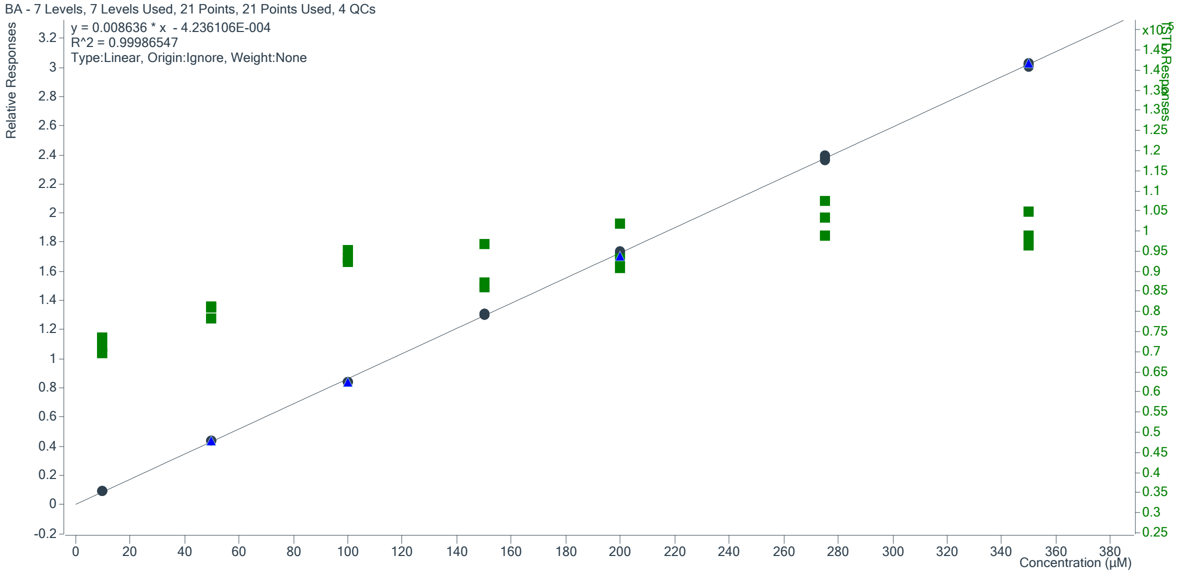

4 – Valeric acid

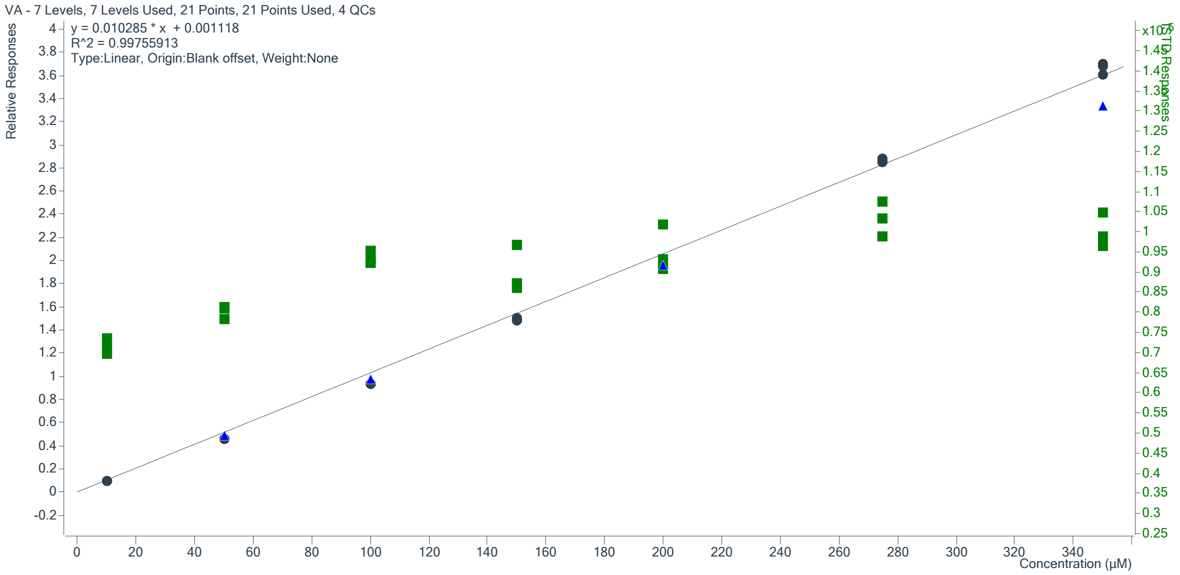

Figure S8. Example chromatograms for SCFA in feces of blanks and samples.

1 – Samples

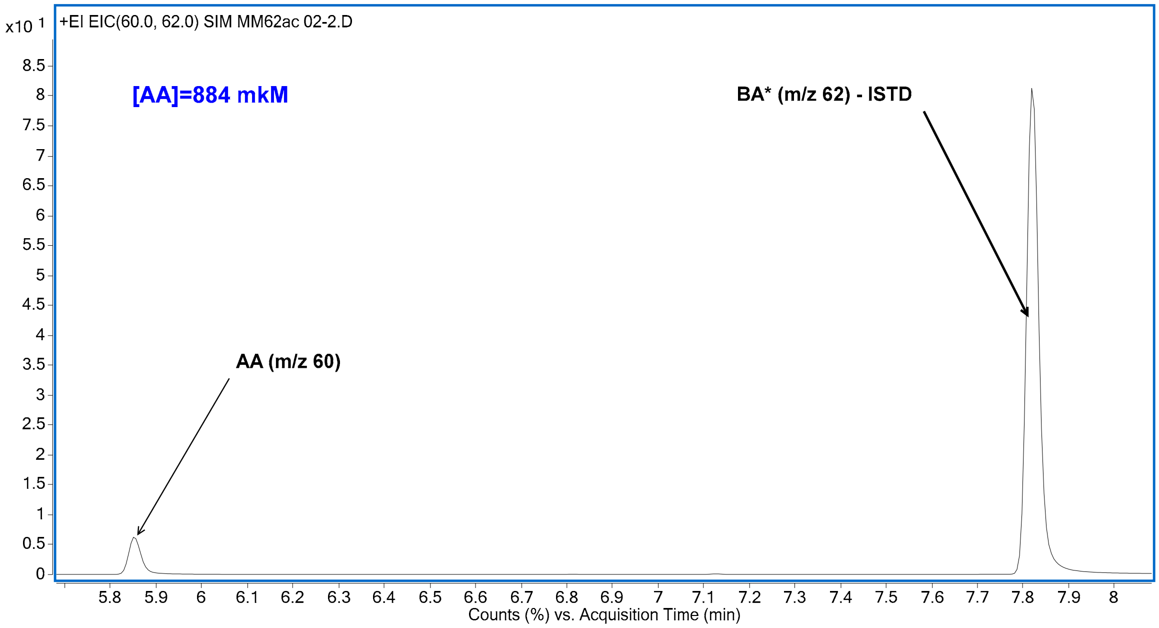

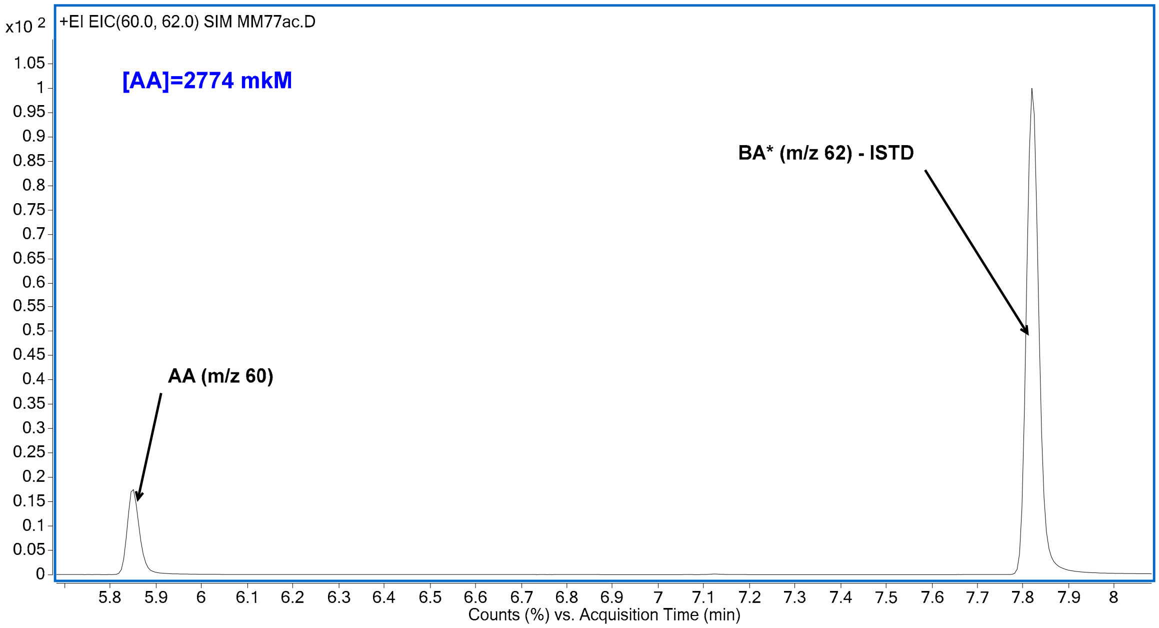

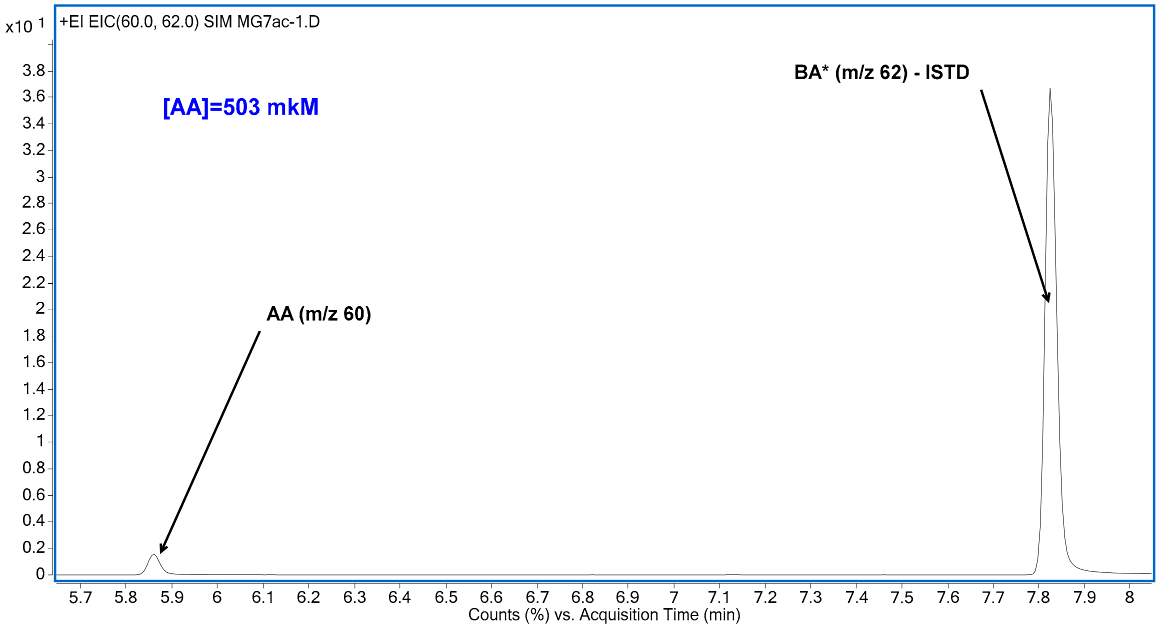

x10 1
+EI EIC(60.0, 62.0) SIM MG7ac-1.D
[AA]=503 mkM
BA* (m/z 62) - ISTD
AA (m/z 60)
3.8
3.6
3.4
3.2
3
2.8
2.6
2.4
2.2
2
1.8
1.6
1.4
1.2
1
0.8
0.6
0.4
0.2
0
5.7 5.8 5.9 6 6.1 6.2 6.3 6.4 6.5 6.6 6.7 6.8 6.9 7 7.1 7.2 7.3 7.4 7.5 7.6 7.7 7.8 7.9 8
Counts (%) vs. Acquisition Time (min)

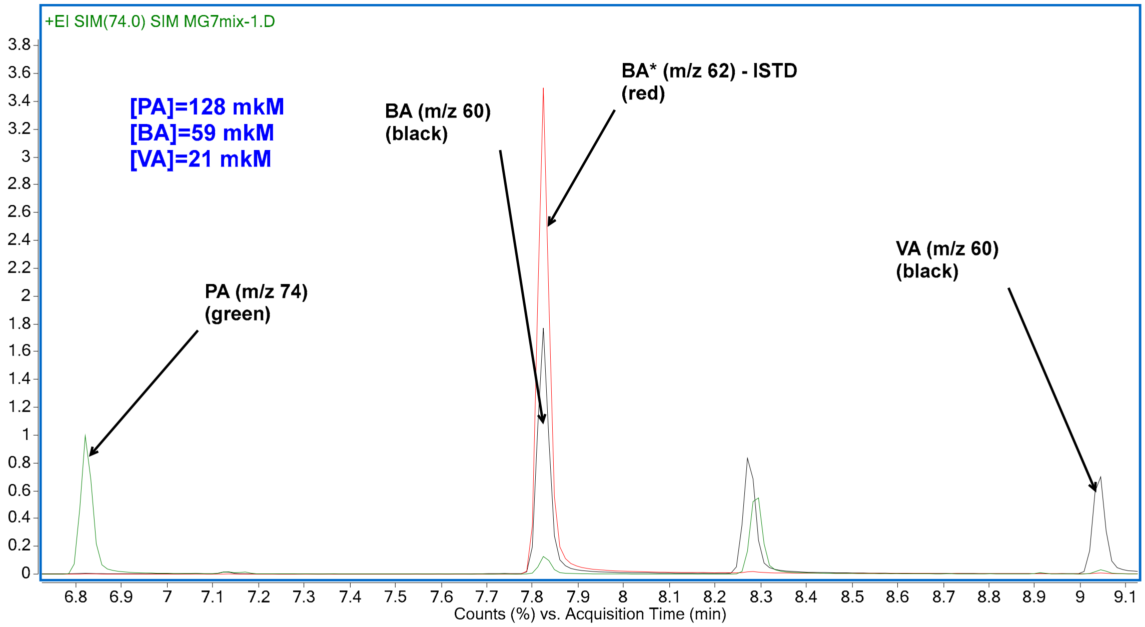

+EI SIM(74.0) SIM MG7mix-1.D
[PA]=128 mkM
[BA]=59 mkM
[VA]=21 mkM
BA (m/z 60)
(black)
BA* (m/z 62) - ISTD
(red)
PA (m/z 74)
(green)
VA (m/z 60)
(black)
3.8
3.6
3.4
3.2
3
2.8
2.6
2.4
2.2
2
1.8
1.6
1.4
1.2
1
0.8
0.6
0.4
0.2
0
6.8 6.9 7 7.1 7.2 7.3 7.4 7.5 7.6 7.7 7.8 7.9 8 8.1 8.2 8.3 8.4 8.5 8.6 8.7 8.8 8.9 9 9.1
Counts (%) vs. Acquisition Time (min)

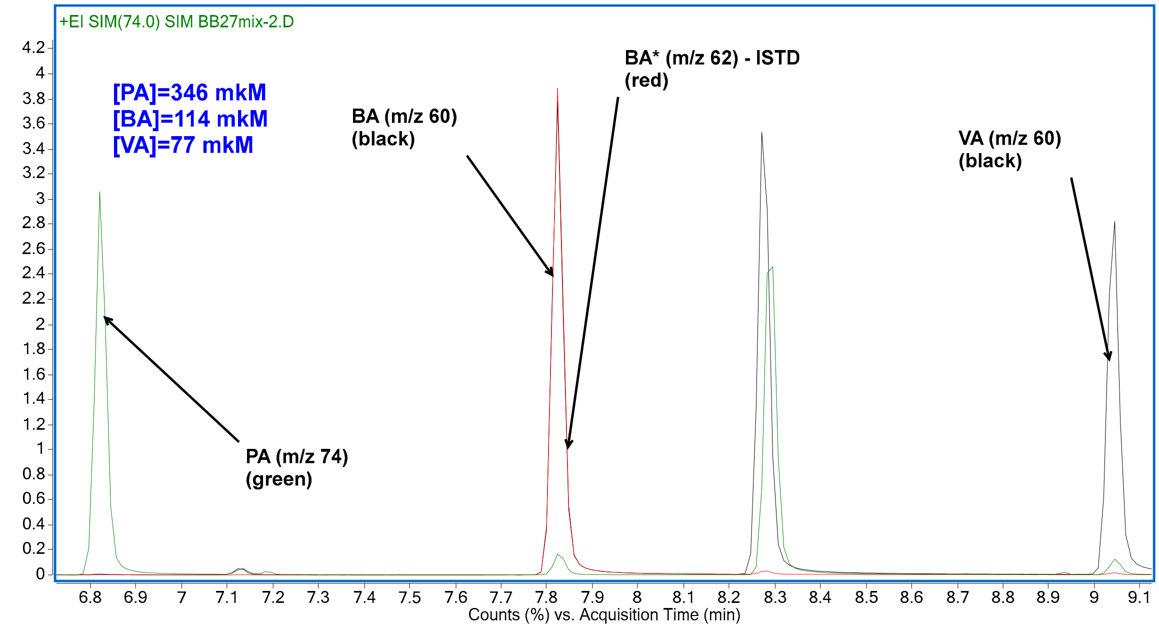
+EI SIM(74.0) SIM BB27mix-2.D
[PA]=346 mkM
[BA]=114 mkM
[VA]=77 mkM
BA (m/z 60)
(black)
BA* (m/z 62) - ISTD
(red)
VA (m/z 60)
(black)
PA (m/z 74)
(green)
Counts (%) vs. Acquisition Time (min)

2 – Blank

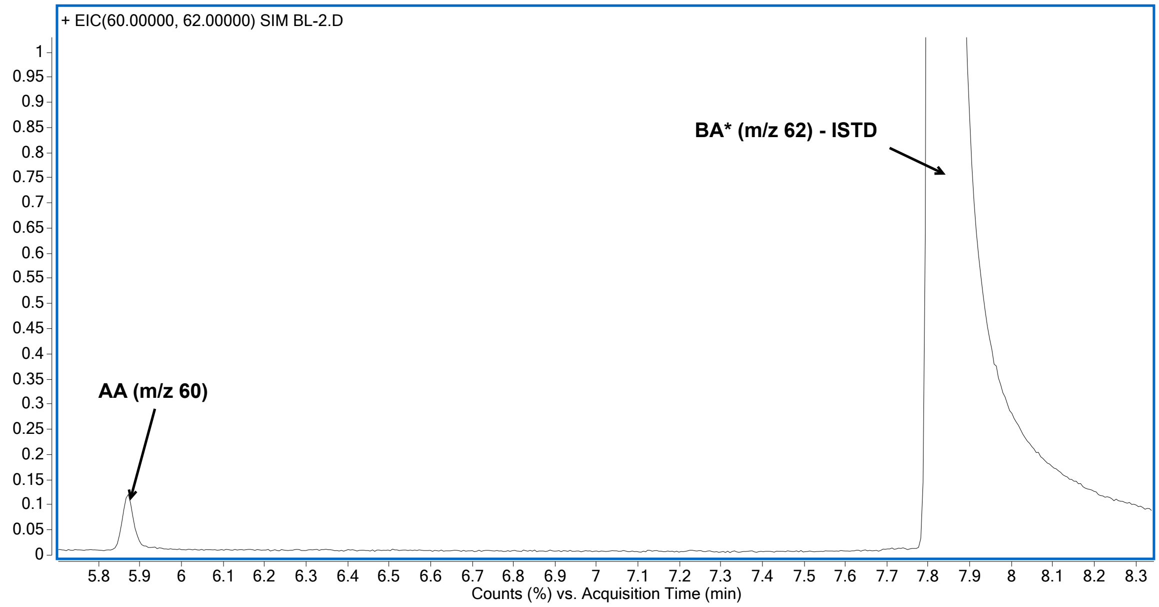
+ EIC(60.00000, 62.00000) SIM BL-2.D
BA* (m/z 62) - ISTD
AA (m/z 60)
Counts (%) vs. Acquisition Time (min)

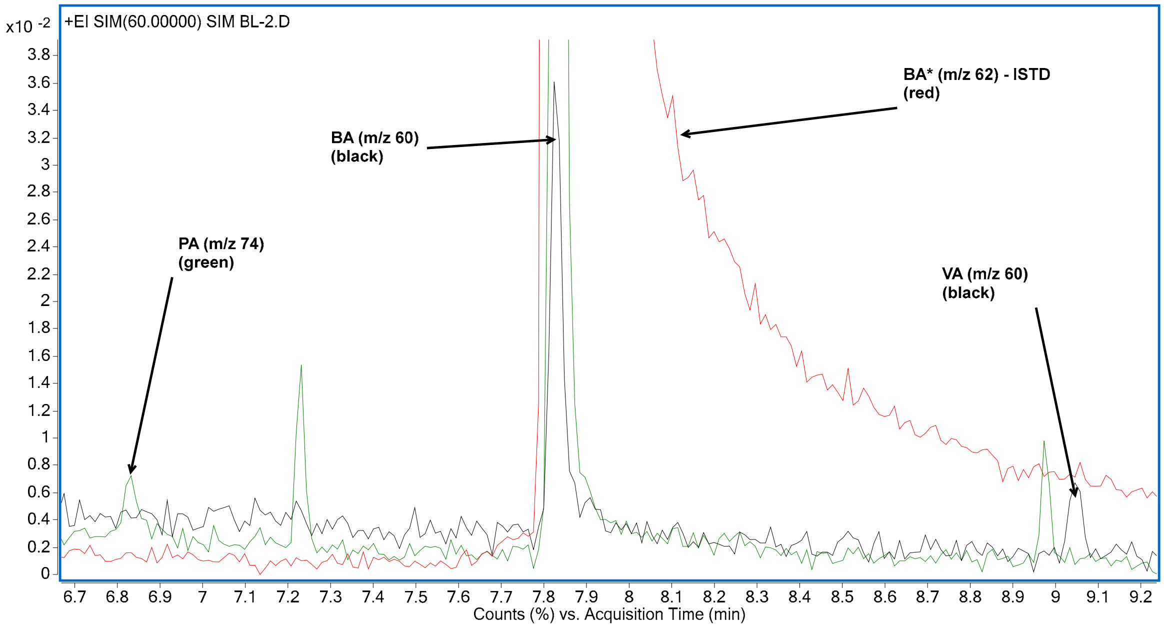

x10 -2
+EI SIM(60.00000) SIM BL-2.D
BA* (m/z 62) - ISTD
(red)
BA (m/z 60)
(black)
PA (m/z 74)
(green)
VA (m/z 60)
(black)
3.8
3.6
3.4
3.2
3
2.8
2.6
2.4
2.2
2
1.8
1.6
1.4
1.2
1
0.8
0.6
0.4
0.2
0
6.7 6.8 6.9 7 7.1 7.2 7.3 7.4 7.5 7.6 7.7 7.8 7.9 8 8.1 8.2 8.3 8.4 8.5 8.6 8.7 8.8 8.9 9 9.1 9.2
Counts (%) vs. Acquisition Time (min)

3 – Calibration levels

[AA]=500 μM

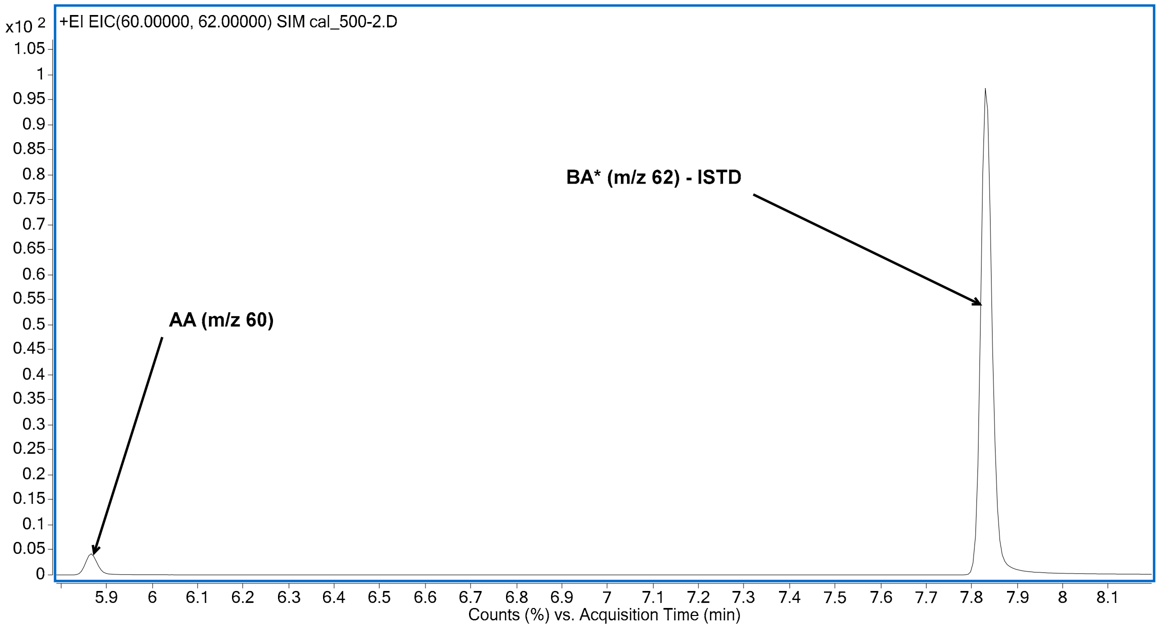

[PA]= [BA]= [VA]=50 μM

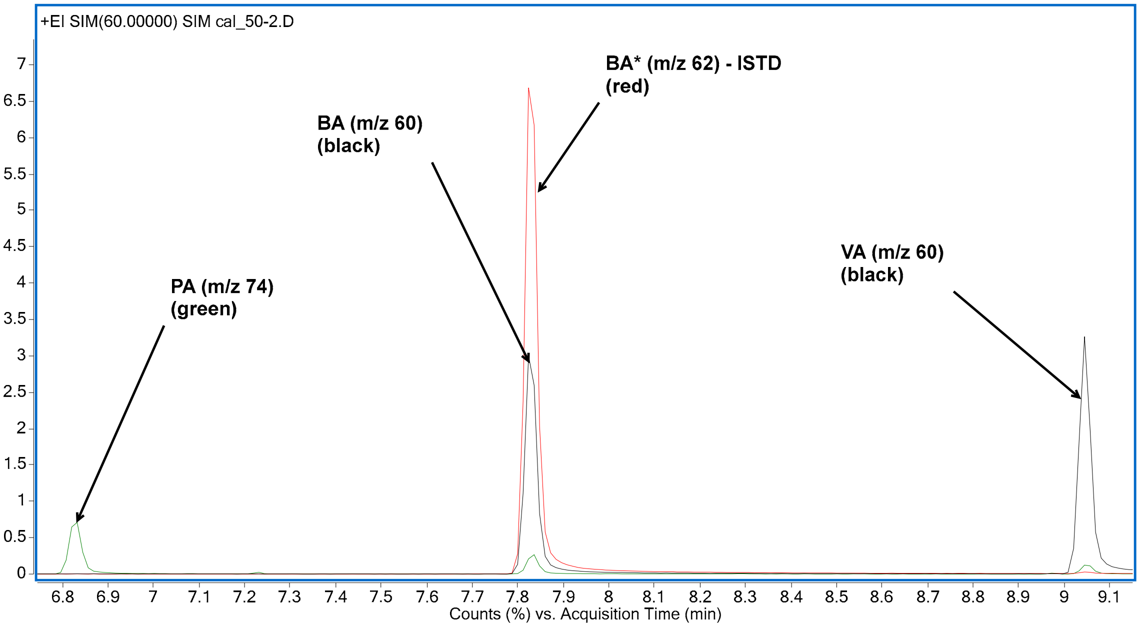

[AA]=3500 μM

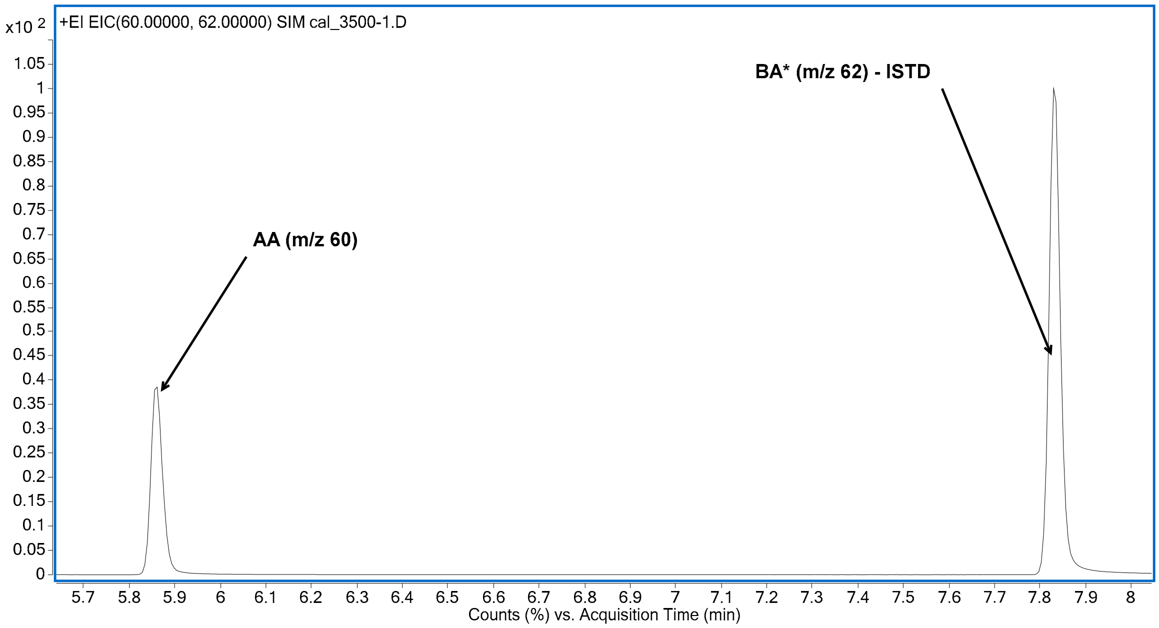

[PA]= [BA]= [VA]=350 μM

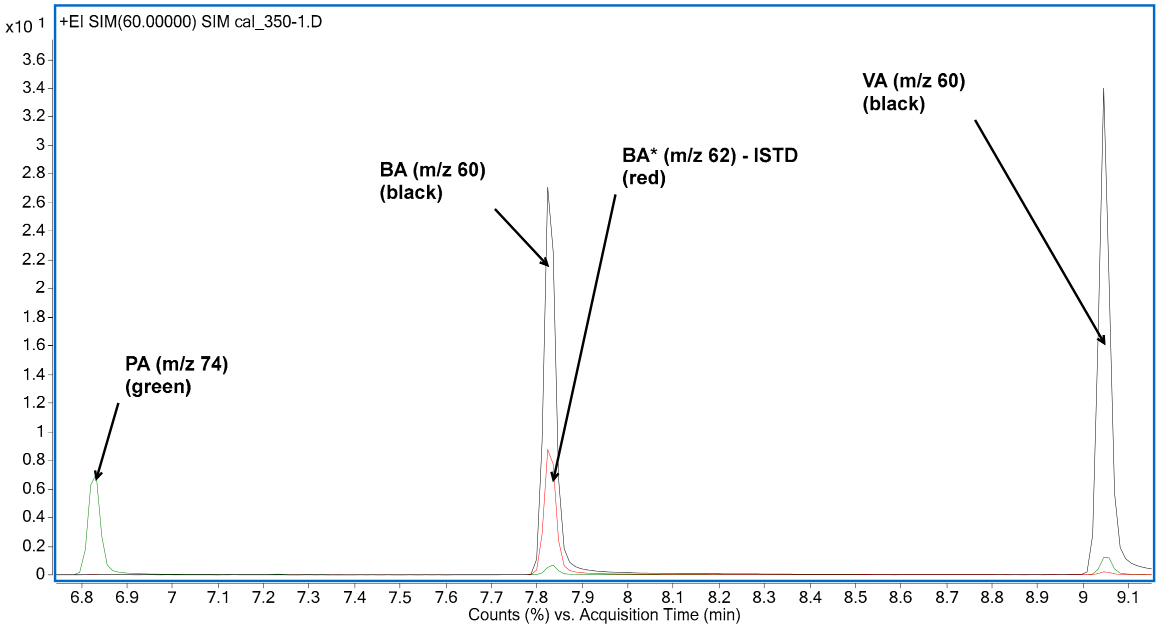

Figure S9. Calibration curves for SCFAs in blood plasma. Milli-Q water was used as matrix. Black circle – standard, green square – IS, blue rectangle – QC.

1 – Acetic acid

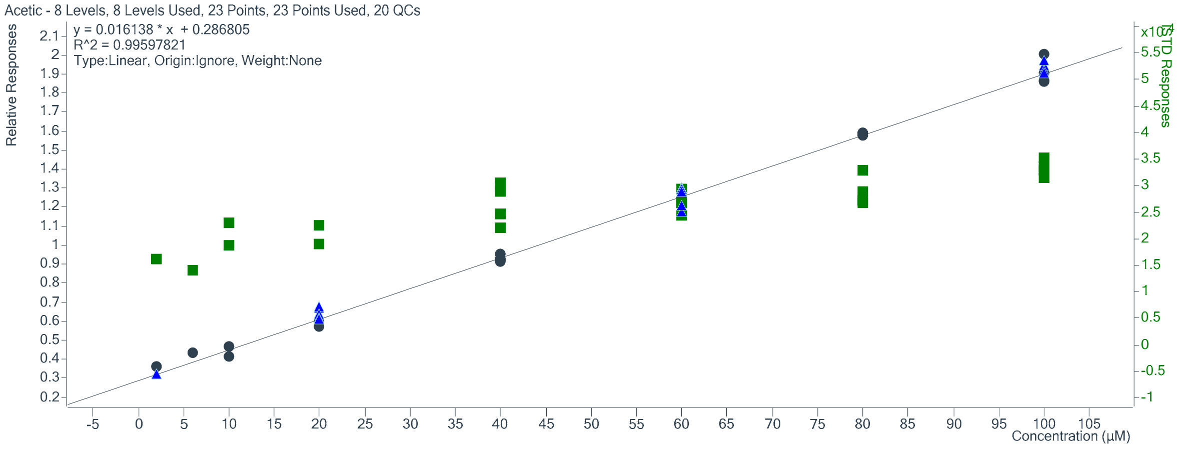

2 – Propionic acid

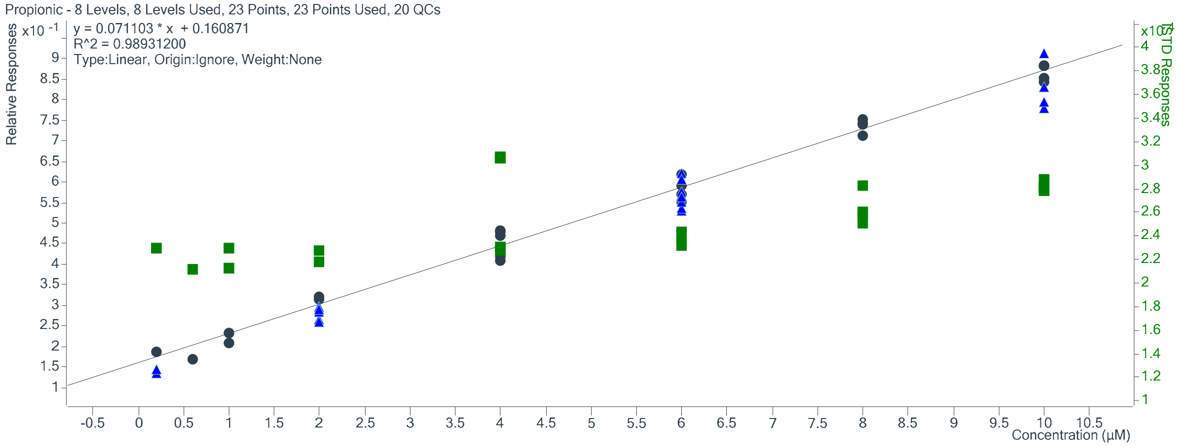

3 – Butyric acid

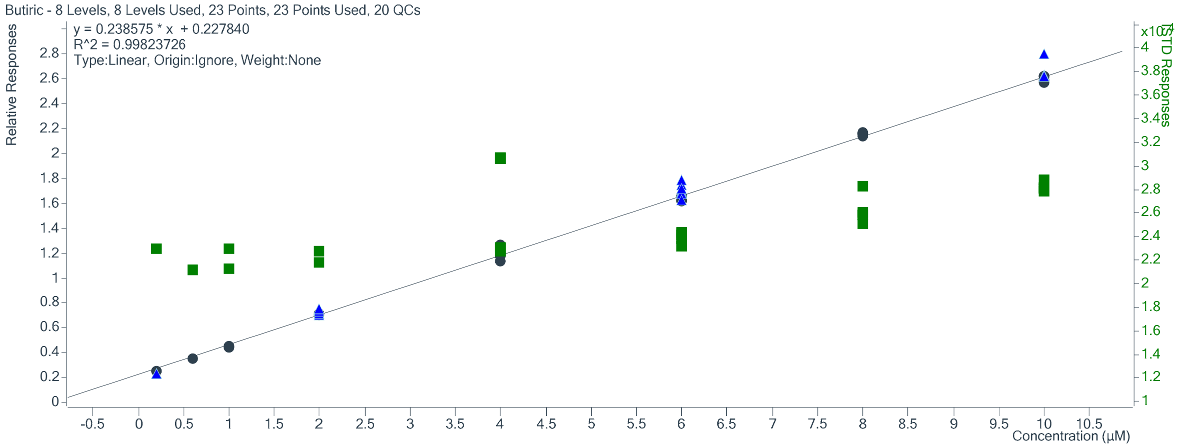

4 – Valeric acid

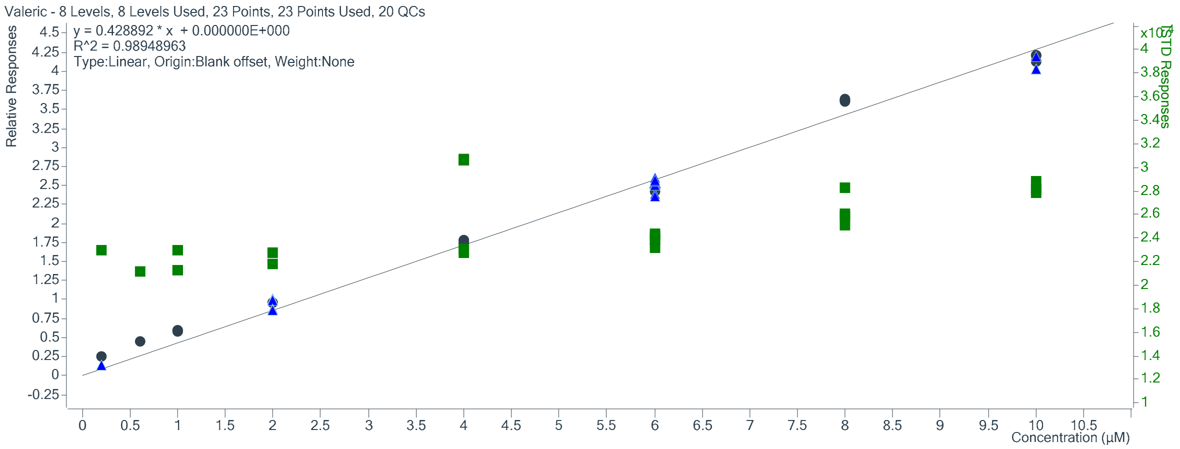

Figure S10. Example chromatograms for SCFA in serum of blanks and samples.

1 – Samples

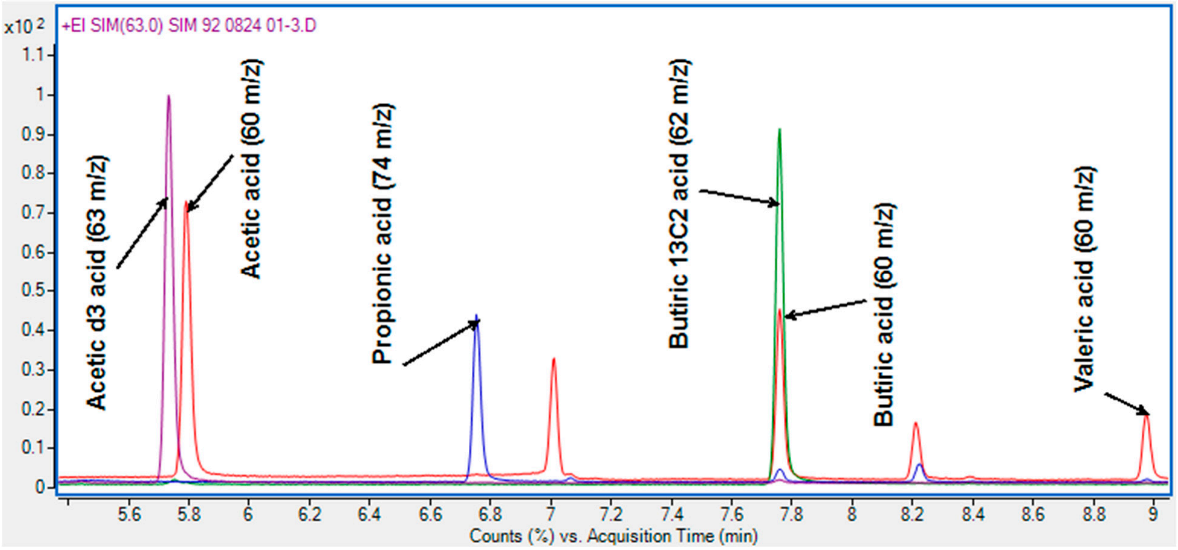

2 – Blank

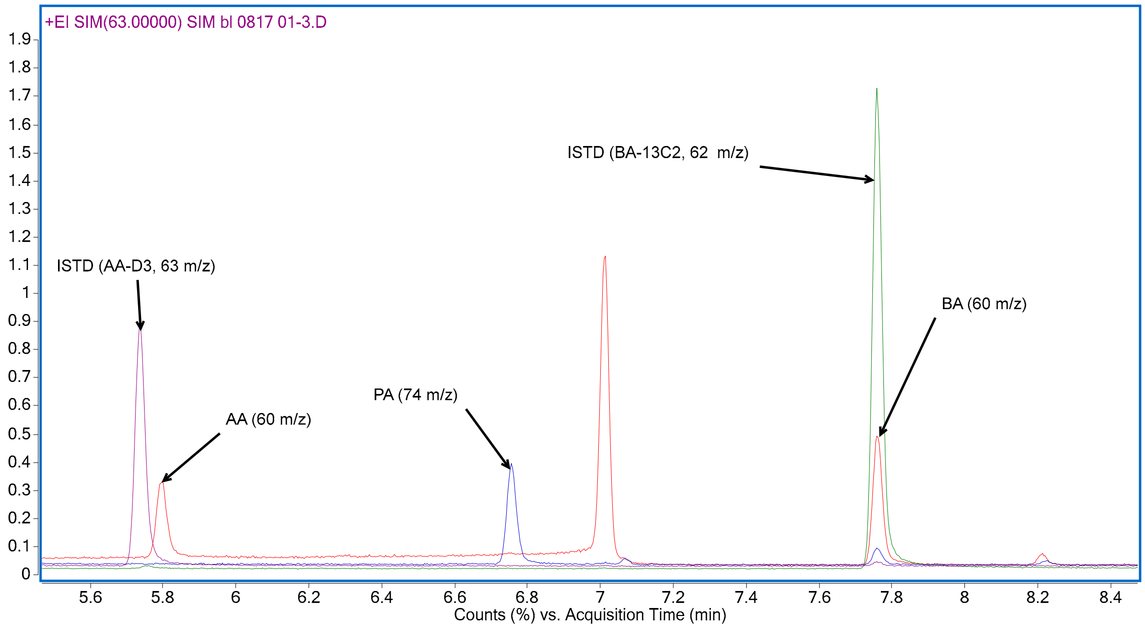

3 – Calibration levels

Low level [AA]=2 μM, [PA]= [BA]= [VA]=0.2 μM

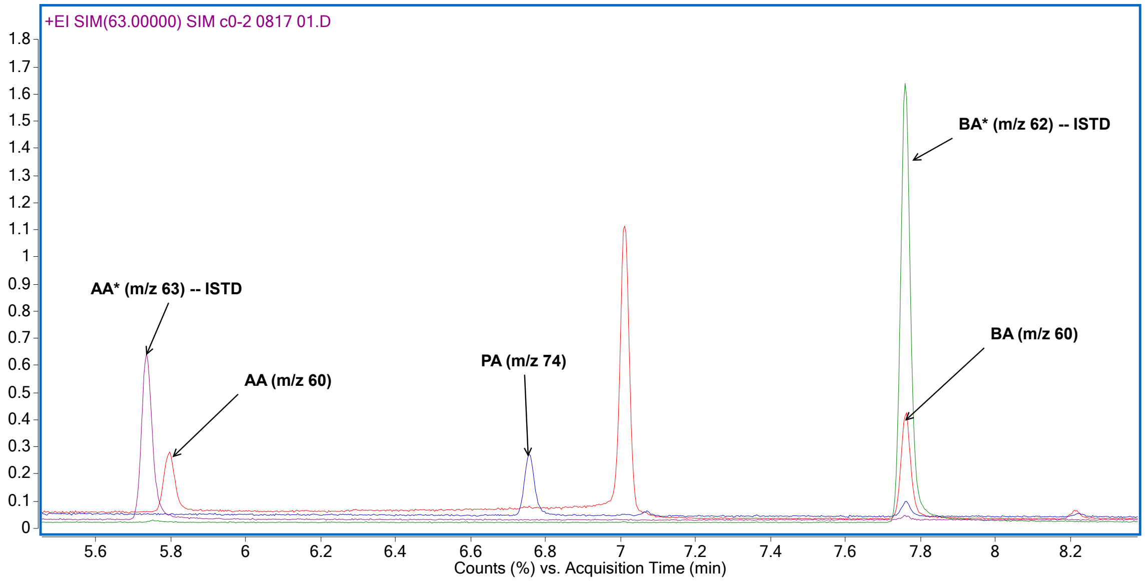

Table S1. Body composition that have statistically significant associations with clinical parameters: coefficient of correlation (R), confidence interval of correlation (CI(R)) and level of significance (P).

Body composition	Clinical parameter	R	CI R	P
	BMI	0.89	0.80-0.94	<0.001
	Android fat, g	0.93	0.87-0.96	<0.001
	Gynoid fat, g	0.78	0.60-0.88	<0.001
	Total fat, %	0.97	0.94-0.98	<0.001
	Total fat, g	0.49	0.19-0.70	0.003
	Visceral fat, g	0.9	0.82-0.95	<0.001
	SHBG	-0.46	-0.68--0.15	0.006
	Androstendion	-0.54	-0.74--0.25	0.001
	Glucose (fasting)	0.34	0.02-0.60	0.04
	Insulin (fasting)	0.57	0.30-0.76	<0.001
	Triglycerides	0.51	0.22-0.72	0.001
	LDL	0.36	0.04-0.62	0.031764
	HDL	-0.55	-0.74--0.27	<0.001
	IL-6	0.54	0.25-0.73	0.001
Body fat, kg	CRP	0.55	0.28-0.75	<0.001
	BMI	0.86	0.77-0.92	<0.001
	Body fat, kg	0.90	0.84-0.94	<0.001
	Android fat, g	0.85	0.75-0.91	<0.001
	Ginod fat, g	0.66	0.48-0.79	<0.001
	Total fat, %	0.87	0.79-0.93	<0.001
	Total fat, g	0.50	0.27-0.68	0.003
	SHBG	-0.58	-0.73--0.37	<0.001
	Androstendion	-0.40	-0.60--0.15	0.003
	Glucose (fasting)	0.35	0.09-0.56	0.009
	Insulin (fasting)	0.57	0.36-0.73	<0.001
	Insulin (after 1 hour)	0.36	0.11-0.57	0.007
	Insulin (after 2 hours)	0.36	0.11-0.57	0.007
	Cholesterol	0.29	0.03-0.52	0.03
	Triglycerides	0.58	0.37-0.73	<0.001
	LDL	0.35	0.09-0.56	0.02
	HDL	-0.52	-0.69--0.29	<0.001
	IL-6	0.52	0.30-0.69	<0.001
	TNF-α	0.32	0.06-0.54	0.04
Visceral fat, g	CRP	0.49	0.26-0.67	<0.001
	BMI	0.87	0.79-0.92	<0.001
	Body fat, kg	0.97	0.95-0.98	<0.001
	Android fat, g	0.93	0.89-0.96	<0.001
	Gynoid fat, g	0.87	0.80-0.92	<0.001
	Total fat, g	0.51	0.31-0.67	0.002
	Visceral fat, g	0.87	0.80-0.92	<0.001
	SHBG	-0.54	-0.69--0.35	<0.001
	Androstendion	-0.36	-0.55--0.14	0.003
	Insulin (fasting)	0.51	0.31-0.67	<0.001
	Insulin (after 1 hour)	0.38	0.16-0.57	0.002
Total fat, %	Insulin (after 2 hours)	0.30	0.07-0.50	0.01

	Cholesterol	0.29	0.06-0.5	0.02
	Triglycerides	0.47	0.26-0.63	<0.001
	LDL	0.34	0.11-0.54	0.009
	HDL	-0.39	-0.57--0.16	0.003
	IL-6	0.55	0.36-0.70	<0.001
	TNF-α	0.29	0.06-0.49	0.03
	CRP	0.44	0.22-0.61	<0.001
	BMI	0.61	0.35-0.79	<0.001
	Body fat ,kg	0.49	0.18-0.71	0.003
	Android fat, g	0.45	0.13-0.68	0.008
	Ginod fat, g	0.46	0.14-0.69	0.007
	Total fat, %	0.51	0.21-0.72	0.002
	Visceral fat, g	0.50	0.20-0.72	0.003
Total fat, g	HDL	-0.41	-0.66--0.08	0.02

Table S2. **Abundance of gut bacteria** with statistically significant alteration between control group and PCOS group before treatment (logarithmic values): levels of significance (P), and fold change of the median (FC).

**Microorganism**	**Control**	**POCS**	**P**	**FC**	**׀Effect size׀**	**Power**
Erysipelotrichaceae	7.6(7.3;7.9)	7.1(6.6;7.6)	0.02	0.32	0.70	0.66
Prevotella spp	6.8(5.9;7.8)	4.9(3.8;7.4)	0.02	0.01	<0.001	0.05
Clostridium perfringens gr	5.4(5.2;5.8)	4.8(3.8;5.4)	0.02	0.25	0.06	0.06
Clostridium leptum gr	9.4(9.1;9.6)	9.1(8.8;9.4)	0.03	0.50	0.74	0.74

Table S3. Concentrations of fecal SCFAs with statistically significant alterations between the control group and the PCOS group prior to treatment: levels of significance (P) and fold change of median (FC).

**SCFA**	**Control**	**PCOS**	**P**	**FC**	**׀Effect size׀**	**Power**
Acetic acid, μM	2040(1664;2754)	2998(2009;3573)	0.04	1.47	0.36	0.27
Valeric acid, μM	82(67;123)	126(78;166)	0.048	1.54	0.47	0.42

Table S4. Clinical parameters that have statistically significant associations with the concentration of serum SCFAs: coefficient of correlation (R), confidence interval of correlation (CI(R)) and level of significance (P).

**Parameter**	**SCFAs**	**R**	**CI (R)**	**P**
BMI	Acetic acid, μM	-0.33	-0.56--0.048	0.02
Total fat, %	Acetic acid, μM	-0.36	-0.61--0.05	0.03
Visceral fat, g	Acetic acid, μM	-0.36	-0.64--0.002	0.049
	Acetic acid, μM	-0.41	-0.65--0.10	0.01
	Acetic acid, rel. part	-0.58	-0.76--0.30	<0.001
	Propionic acid, rel. part	0.57	0.30-0.76	0.004
	Butyric acid, rel. part	0.41	0.10-0.65	0.01
Triacylglycerides	Valeric acid, rel. part	0.36	0.03-0.61	0.03
	Acetic acid, rel. part	-0.42	-0.66--0.11	0.009
Cholesterol	Propionic acid, rel. part	0.43	0.12-0.66	0.008
HDL	Acetic acid, μM	0.36	0.005-0.64	0.047
SHBG	Acetic acid, μM	0.37	0.05-0.62	0.03

	Butyric acid, rel. part	-0.33	-0.59--0.01	0.04
	Valeric acid, rel. part	-0.33	-0.59--0.005	0.047
AMH	Valeric acid, rel. part	-0.33	-0.58--0.007	0.05
	Acetic acid, μM	-0.52	-0.72--0.23	0.001
	Propionic acid, μM	-0.46	-0.69--0.16	0.004
	Butyric acid, μM	-0.49	-0.71--0.20	0.002
TNF-α	Valeric acid, μM	-0.39	-0.63--0.07	0.02
IL-6	Acetic acid, μM	-0.51	-0.71--0.21	0.002
	Propionic acid, μM	0.33	0.01-0.59	0.04
Glucose (fasting)	Valeric acid, μM	0.32	0.006-0.58	0.047
	Acetic acid, rel. part	-0.40	-0.64--0.08	0.01
Glucose (after 1 hour)	Propionic acid, rel. part	0.39	0.08-0.64	0.02
	Acetic acid, μM	-0.39	-0.64--0.07	0.02
Insulin (after 1 hour)	Valeric acid, rel. part	0.33	0.005-0.6	0.048

Table S5. Reference ranges for laboratory parameters.

Parameter	Reference range
Cholesterol (mmol/l)	3,1-5,2
Triacylglycerides (mmol/l)	0,7-1,7
HDL (mmol/l)	0,9-1,8
LDL (mmol/l)	0-3,9
Atherogenicity index	2,0-3,0
Fasting glucose (mmol/l)	3,9-5,6
Glucose two hours after drinking the glucose solution (mmol/l)	<7,8
Fasting insulin, mcIU/ml	2,7-25
AMG, ng/ml	0,03–7,37
LH, mIU/ml	1,68–15,0
FSH, mIU/ml	1,37–9,9
Prolactin, mIU/l	109–557
17-OHP, nmol/l	1,24–8,24
TSH, mIU/l	0,4–4,0
Total testosterone, nmol/l	0,38-1,72
Free testosterone, ng/ml	<2,85
Androstenedione, nmol/l	1,6-19,0
Index of free testosterone	0,7 - 8,7%
PSSG, nmol/ml	18,0-114,0
